# Responses of Rhizosphere Bacterial and Fungal Communities to the Long-Term Continuous Monoculture of Water Oat

**DOI:** 10.3390/microorganisms10112174

**Published:** 2022-11-02

**Authors:** Gang Wu, Feifei Yu, Manman Yuan, Jiabao Wang, Chuang Liu, Weizhu He, Zhihuan Ge, Yixiang Sun, Yuan Liu

**Affiliations:** 1 Institute of Soil and Fertilizer, Anhui Academy of Agricultural Sciences, Hefei 230031, China; 2 Key Laboratory of Nutrient Cyclling and Resources Environment of Anhui Province, Hefei 230031, China; 3 Institute of Horticulture, Anhui Academy of Agricultural Sciences, Hefei 230031, China; 4 College of Life Science, Huaibei Normal University, Huaibei 235000, China

**Keywords:** water oat, continuous planting, enzyme activity, rhizosphere soil, microbial community

## Abstract

As an cultivated aquatic vegetable, the long-term continuous monocropping of water oat results in the frequent occurrence of diseases, the deterioration of ecological system and decreased quality of water oat. In this study, real-time quantitative PCR (qPCR) and Illumina high-throughput sequencing were used to determine the dynamic changes in bacterial and fungal communities in rhizosphere soil under continuous cropping of water oat for 1, 5, 10, 15 and 20 years (Y1, Y5, Y10, Y15 and Y20), and soil properties and enzyme activities were also determined. Results showed that the contents of soil organic carbon (SOC), total nitrogen (TN), alkali-hydrolyzable nitrogen (AN), available phosphorus (AP) and the activities of four soil enzymes increased in Y5 and Y10 and then decreased in Y15 and Y20. Spearman correlation analysis identified SOC, TN, AP and AN as the main factors that affect the four enzyme activities. The qPCR results showed that there was no significant difference in bacterial abundance between the different planting years, while the fungal abundance first increased and then decreased. The long-term continuous planting of water oat (Y15 and Y20) significantly reduced the operational taxonomic unit numbers and the Shannon, Chao1, and ACE indices of rhizosphere bacteria and fungi. The bacterial and fungal community compositions were markedly affected by the continuous planting year. The relative abundances of Bacteroidetes and Firmicutes decreased significantly in Y10 and Bacteroidetes increased significantly in Y15. Relative abundances of dominated Mortierellomycota and Ascomycota phyla increased with the continuous cropping years, while Rozellomycota presented the opposite trend. The AK, AN, and SOC were the main factors that changed the bacterial community, while AK and AP significantly shifted the fungal community. Thus, long-term continuous planting of water oat resulted in the deterioration of soil nutrients and microbial communities. The results provided a reference for the remediation of soil under continuous water oat planting and sustainable development of water oat industry.

## 1. Introduction

Water oat, a perennial herb of the *Zizania* genus in the grass family, is widely planted in southeast Asian countries, such as China, Japan, and South Korea. It has an annual planting area of about 70,000 hectares in China [1]. As the second most important cultivated aquatic vegetable in China, it ranks second place among all the vegetables in China in terms of nutritional and healthcare values. Its cultivation has important economic significance [2]. However, the long-term continuous planting of water oat can lead to the accumulation of pathogenic bacteria, which causes a decrease in the quality of water oat and soil [3]. The existing studies on water oat mostly focus on its yield, quality, storage, and nutritional and healthcare values [2,4] but rarely explore whether the long-term planting of water oat would cause soil degradation and change the soil microbial structures. In this context, studying the changes in the soil environment of water oat with different planting years is of great significance for understanding and evaluating the effect of water oat on soil quality and the sustainable development of water oat.

Soil microbes constitute an essential part of soil ecosystems and are the main participants in soil nutrient cycling and energy flow and play a vital role in soil structure improvement, fertility maintenance, environmental pollutant purification, and plant growth [5,6,7]. Since they are directly involved in the decomposition of soil organic matter, they not only directly affect the soil nutrient contents and soil enzyme activity but also indirectly influence the growth of aboveground plants. In this sense, they constitute an important indicator of soil quality [8,9]. Studies have shown that different planting years have varying effects on soil nutrient contents, but long-term continuous cropping will not only reduce the contents of available phosphorus (AP) and available potassium (AK) but also significantly lower the α-diversity indices of soil microbes and weaken the ability of soil microbes to utilize carbon sources [10,11,12]. Studies have revealed that with increasing planting years, the total amounts of rhizosphere soil microbes of tea trees (*Camellia sinensis*), grapes (*Vitis vinifera*), and vegetables first increase and then decrease [1,13,14]. Liu et al. (2021) conducted field experiments that showed that compared with other short-term planting years, a continuous planting period of 20 years significantly reduced the soil bacterial and fungal community diversity and abundance of grapes, and that AK and alkali-hydrolyzable nitrogen (AN) were the main soil factors associated with the transfer of bacterial and fungal communities [15]. Zhu et al. (2019) showed that the microbial functional diversity and community structure of rhizosphere soil under the long-term planting of kiwifruit (*Actinidia deliciosa*) decreased [16]. Wang et al. (2022) found that long-term continuous cropping significantly increased the abundance of fungal communities and changed the distribution of bacterial and fungal communities, causing changes in community diversity [17]. Some studies have also found that the soil microbial abundance and diversity increased with increasing planting years [18,19]. In summary, the effects of continuous planting on the microbial diversity and structure in rhizosphere soil vary with plants. However, few studies have evaluated response of soil microbial communities and enzyme activities to water oat replanting. Therefore, exploring the effects of different planting years on the microbial community of water oat is of great significance for the scientific fertilization and ecological improvement of the areas that are suitable for water oat growth.

Soil microbes produce large quantities of enzymes, which play an important role in the decomposition and mineralization of soil organic matter and the cycling of soil nutrients [20]. It has been proven that when the soil is disturbed, enzyme activity is one of the properties of soil that is the most affected, thus, soil enzyme activity is considered a good indicator of soil quality [21,22]. However, studies are in conflict when it comes to the effects of different planting years on soil enzymes. Lou et al. (2007) studied the activities of enzymes in soil under different tobacco (*Nicotiana tabacum*) planting years and showed that the activities of catalase, invertase, urease, and neutral phosphatase presented a parabolic change, i.e., first decreasing and then increasing, with the increase in continuous cropping years [23]. Guo et al. (2010) studied the soil enzyme activity of navel orange (*Citrus sinensis*) orchards and found that with the extension of fertilization time, the activity of soil sucrase first increased and then decreased, while that of acid phosphatase increased [24]. Some studies have also shown that the activities of different soil enzymes change in varying manners with different planting years, and there is a significant correlation between soil enzymes and soil physicochemical properties [25,26]. Thus, investigating the patterns of change and interactions of soil microbes and soil enzyme activity with different planting years of water oat can provide a theoretical basis for evaluating the scientific production of water oat.

Taking the soil of water oat from different planting years as the research object, this study examined its physicochemical properties, enzyme activities, and microbial community diversity, analyzed the change in characteristics of the soil properties, enzyme activity, and microbial community structure and abundance, and revealed the change in quality and microbial driving mechanism of the soil under the long-term planting of water oat, thereby laying a scientific foundation for the cultivation and production of water oat and the sustainable development of soil.

## 2. Materials and Methods

### 2.1. Site Description and Soil Sampling

The experimental soil was paddy soil collected from Zhubu Town (116°27′ N and 31°03′ E) and Shiguan Township (116°23′ N and 30°59′ E) of Yuexi County, Anhui Province, China. The study area belongs to a north subtropical humid monsoon climate region with a complex distribution of mountain vertical natural belts and large microclimatic differences. It has an average annual temperature of 14.4 °C, a temperature range of 2–26.3 °C and an average annual rainfall of 1103.2 mm. Water oat (Lu’an water oat) was transplanted in March and harvested in September in the growing season. Rhizosphere soil was sampled on 23 September 2021 (at the ripening stage), one week before harvesting. Soils were sampled from water oat of continuous replanting for 1, 5, 10, 15 and 20 years (Y1, Y5, Y10, Y15 and Y20). Each treatment had three replicates. For soil sampling, each plot randomly collected soil samples of five individual water oat plants and mixed them into one replicate. After shaking off the loosely adhered soil of the roots, the remaining soil (about 1 cm thick soils) was collected and homogenized to form on a rhizosphere soil sample. Soil samples were sieved through a 2-mm sieve and immediately shipped to the laboratory in plastic ice bags. A portion of soil was stored at 4 °C for soil enzyme activity and DNA extraction within one week. A portion was air-dried at room temperature to determine the soil properties.

### 2.2. Soil Properties Analyses

Soil pH was measured using a pH meter in a 1:2.5 soil-water suspension. The soil AN was determined using the alkali hydrolysis diffusion method [27]. The soil organic carbon (SOC) was determined by the potassium dichromate external heating method, and total nitrogen (TN) was determined using the Kjeldahl nitrogen process [28]. The soil AK and AP were determined by ammonium acetate extraction-flame photometry and sodium bicarbonate extraction-molybdenum antimony spectrophotometry, respectively [29].

### 2.3. Soil Enzyme Activity Analyses

The activity of soil catalase was determined using potassium permanganate titration. The activity of soil urease was determined by sodium phenol–sodium hypochlorite colorimetry. The activity of soil sucrase was determined by 3,5–dinitrosalicylic acid colorimetry. The activity of soil alkaline phosphatase was determined by disodium phenyl phosphate colorimetry [30].

### 2.4. Soil DNA Extraction and Real-Time Quantitative PCR

Total DNA from the soil was extracted from 0.5 g of fresh soil using an SPINeasy soil DNA kit (MP Biomedicals, LLC, USA). The DNA quality was tested by 1% agarose gel electrophoresis and an ultra-micro-UV spectrophotometer (Bio-Rad Laboratories Inc., Hercules, CA, USA).

The soil bacterial and fungal gene abundances were both determined by real-time quantitative PCR (qPCR). The primers 338F/518R (338F-ACTCCTACGGGAGGCAGCAG and 518R-ATTACCGCGGC TGCTGG) and ITS1F/ITS2R (ITS1F-CTTGGTCATTTAGAGGAAGTAA and ITS2R-GCTGCGTTCT TCATCGATGC) were used to amplify the bacterial 16S rRNA and fungal ITS genes, respectively [17,31]. The amplified products were purified and cloned into the PMD18-T vector. The positive clones were screened, followed by their culture and the extraction of plasmids. The concentrations of plasmids were measured using an ND-1000, and the gene copy numbers were calculated. The 10-fold serial dilutions of plasmids with a known copy number were used as DNA templates for the standard curve. qPCR was performed in a Roche LightCycler 480 (Roche Diagnostics, Germany) system. The reaction system included 20 μL:10 μL 2 × Universal SYBR Green Fast qPCR Mix (ABclonal Technology, Woburn, MA, USA); 8 μL sterile ultrapure water; 0.5 μL forward primer and 0.5 μL reverse primer (10 μM); and 1 μL DNA template. Each DNA sample was amplified three times. The dissolution curve was used to analyze the specificity of the amplified products. The amplification efficiency fell between 97% and 106% with R^2^ > 0.98.

### 2.5. MiSeq Sequencing and Data Analysis

The V4-V5 region of the bacterial 16S rRNA gene was amplified using PCR primers 515F (5′-GTGCCAGCMGCCGCGG-3′) and 907R (5′-CCGTCAATTCMTTTRAGTTT-3′) [15], and the ITS region of the transcribed spacer was amplified using the primers ITS1F (5′-CTTGGTCATTTAGAGGAAGTAA-3′) and ITS2R (5′-GCTGCGTTCTTCATCGATGC-3′) [17]. The amplified products were purified with a purification kit and then used to detect the quality of genomic DNA with a NanoDrop 2000 followed by library construction. The sizes of sequencing library inserts were measured using an Agilent 2100 Bioanalyzer (Agilent Technologies Inc., Waltham, MA, USA), and the concentrations of sequencing libraries were accurately quantified. Library sequencing was performed uniformly on an Illumina MiSeq platform (Genesky Bio-Tech Inc., Shanghai, China). The raw sequences were quality-filtered using QIIME software to remove low-quality sequences. The remaining sequences were subjected to chimera detection using Mothur software to remove all the chimeric sequences. The non-chimeric sequences were then clustered into operational taxonomic units (OTUs) with 97% similarity using QIIME software. The representative sequences of OTUs were classified and identified using the BLAST algorithm in GenBank. The bacterial and fungal α-diversity was assessed by calculating the Chao1 indices, observed species numbers, ACE indices, and Shannon indices using Mothur software. We have deposited all raw sequences in the National Center for Biotechnology Information (NCBI) Sequence Reads Archive database PRJNA883442.

### 2.6. Statistical Analysis

A one-way analysis of variance (ANOVA) with Duncan’s test was performed using SPSS 20.0 (IBM Corporation, Armonk, NY, USA) to analyze the differences in the soil properties, gene abundance, alpha diversity and taxonomic relative abundance. Spearman’s correlation was used to assess the relationships between soil properties, enzyme activities and microbial relative abundances. Principal coordinate analysis (PCoA), based on the Bray-Curtis distance, was performed to compare the microbial community profiles among the treatments. Redundancy analysis (RDA) was used to assess the relationship between soil properties and microbial community composition. The PCoA and RDA were performed using the “vegan” package in R (V3.6.2).

## 3. Results

### 3.1. Soil Physicochemical Properties and Enzyme Activities

As shown in Table 1, the contents of SOC, TN, AN, and AP tended to first increase and then decrease during the continuous cropping period. Moreover, the contents of SOC and TN peaked in Y5 (32.17 g·kg^−1^ and 2.97 g·kg^−1^, respectively), and then began to decrease. The amplitudes of changes between Y10, Y15, and Y20 were small. The contents of AN and AP peaked in Y10 at 195.33 mg·kg^−1^ and 72.04 mg·kg^−1^, respectively. The content of AN in Y10 was significantly higher than that during the other planting years. The content of AK decreased with increasing planting years. In particular, the contents of AK in Y5, Y10, Y15, and Y20 decreased by 27.4%, 22.7%, 32.1%, and 81.3% compared with that in Y1, respectively. The pH of soil from continuous cropping of water oat fell within the range of 5.46–5.71, and there was no significant change between different continuous cropping years.

**Figure 1:**
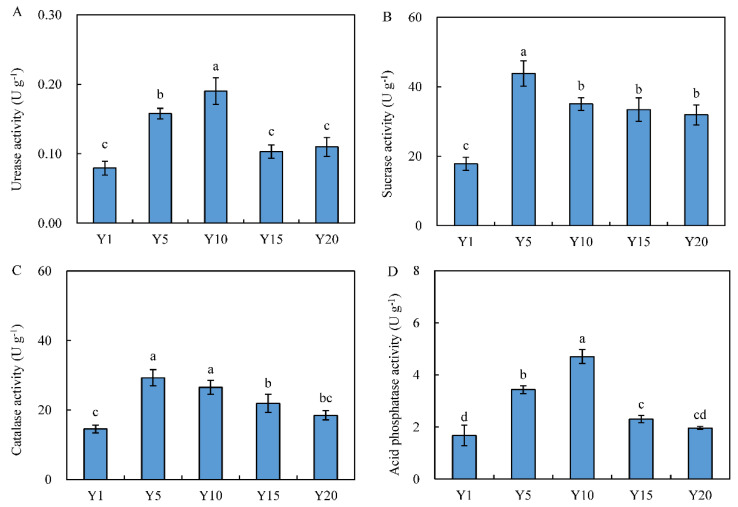
Soil enzyme activities in soil under different planting years of water oat. (**A**) Urease. (**B**) Catalase. (**C**) Sucrase. (**D**) Acid phosphatase. Y1: soil from water oat planting for 1 year. Y5, Y10, Y15 and Y20: soil from continuous water oat replanting for 5, 10, 15 and 20 years, respectively. Different letters indicate significant differences among the treatments at the *p* < 0.05. The error bars indicate the standard error of the means (*n* = 3).

During the continuous planting of water oat, the activity of the four soil enzymes tended to first increase and then decrease (Figure 1). The activities of urease and acid phosphatase peaked after 10 years of continuous cropping, reaching 0.19 U·g^−1^ and 4.70 U·g^−1^, respectively. The activities of sucrase and catalase increased significantly by 54.5% and 50.2% in Y5 compared with those in Y1, respectively. The sucrase activity did not change significantly from Y10 to Y20, while the catalase activity gradually decreased. The correlation between the soil physicochemical properties and soil enzyme activities under different planting years (Table 2) indicated that soil urease and acid phosphatase significantly positively correlated with the soil TN, AP, and AN. Sucrase and catalase significantly positively correlated with the SOC, TN, AP, and AN. Neither the soil pH nor AK had any significant correlation with soil enzyme activity.

### 3.2. Abundance and Diversity of Rhizosphere Bacteria and Fungi

As shown in Table 1, the copy numbers of the bacterial 16S rRNA gene in the rhizosphere soil under different planting years ranged from 2.03 × 10^11^ to 2.92 × 10^11^ copies·g^−1^. There was no significant difference among the planting years. The fungal ITS gene copy numbers of the rhizosphere soil under the different planting years ranged from 1.25 × 10^9^ to 3.01 × 10^9^ copies·g^−1^. Compared with Y1, other planting years increased the fungal gene copy number. Y10 and Y15 significantly increased the fungal copy number by 58.5% and 55.8%, respectively.

**Figure 2:**
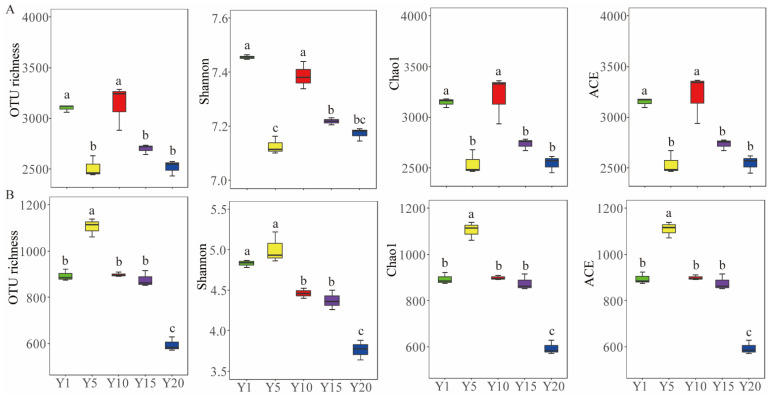
Alpha diversity indices of bacterial (**A**) and fungal (**B**) communities in soil under different planting years of water oat. Y1: soil from water oat planting for 1 year. Y5, Y10, Y15 and Y20: soil from continuous water oat replanting for 5, 10, 15 and 20 years, respectively. Different letters indicate significant differences among the treatments at the *p* < 0.05. The error bars indicate the standard error of the means (*n* = 3).

To study the microbial diversity and community composition in rhizosphere soil from different planting years of water oat, we sequenced the bacterial 16S rRNA and fungal ITS genes and obtained 973,400 and 998,871 effective sequences, respectively. The sequencing depth index coverages of the various samples all exceeded 0.996, indicating that the sequencing amount was sufficient to cover the microbial composition of these samples. The bacterial and fungal OTU numbers were 2446–3287 and 570–1138 at the 97% similarity level, respectively (Figure 2). The α-diversity indices of the bacterial community, including OTU richness, Shannon, Chao1 and ACE, were significantly higher in Y1 and Y10 than those of the other planting years (Figure 2A). The OTU richness, Chao1 and ACE indices of fungal community were significantly increased in Y5 but reduced in Y20 when compared with the Y1 (Figure 2B). The results indicated that the different planting years significantly affected the bacterial diversity rather than bacterial gene abundance in water oat field, and excessively long planting years would lower the fungal diversity and gene abundance.

### 3.3. Communiy Composition of Rhizosphere Bacteria and Fungi

**Figure 3:**
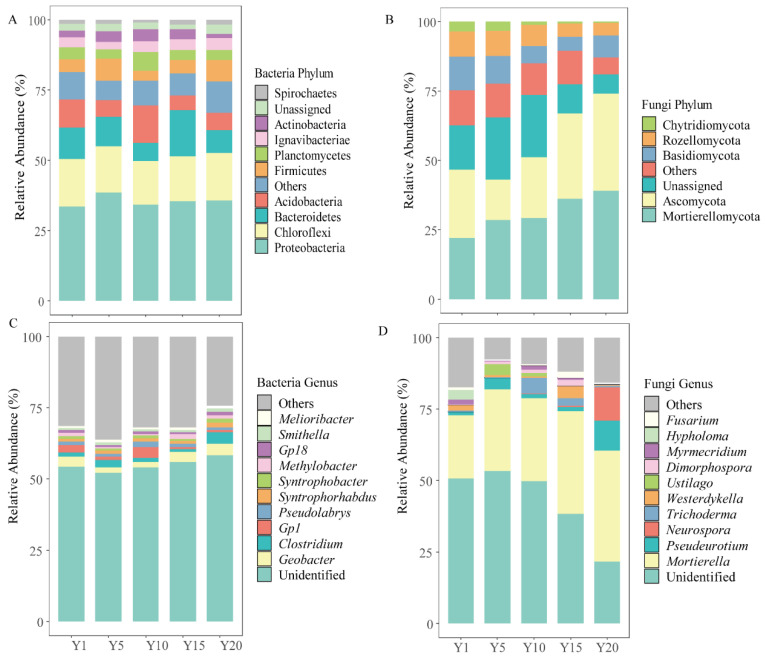
Relative abundances of bacterial and fungal taxa at the phyla (**A**,**B**) and genera (**C**,**D**) levels in soil under different planting years of water oat. Y1: soil from water oat planting for 1 year. Y5, Y10, Y15 and Y20: soil from continuous water oat replanting for 5, 10, 15 and 20 years, respectively.

The microbial community composition in rhizosphere soil under continuous cropping of water oat changed significantly. As shown in Figure 3A, the dominant bacterial phyla in soil under water oat were Proteobacteria (33.52–35.62%), Chloroflexi (15.65–16.82%), Bacteroidetes (6.82–16.42%), Acidobacteria (5.21–13.37%), and Firmicutes (3.62–7.85%). Among them, Proteobacteria and Chloroflexi dominated the bacterial community and remained relatively stable across different planting years. Bacteroidetes decreased significantly in Y10 but increased in Y15. The relative abundance of Acidobacteria increased significantly in Y10, while that of Firmicutes decreased significantly. The dominant fungal phyla in soil under water oat were successively Mortierellomycota (22.18–38.97%), Ascomycota (14.36–35.03%), Basidiomycota (4.97–12.35%), and Rozellomycota (4.47–8.98%) (Figure 3B). Among them, Mortierellomycota and Ascomycota dominated the fungal community. The relative abundances of Mortierellomycota and Ascomycota increased with increasing planting years, while Rozellomycota presented the opposite trend. In contrast, the relative abundance of Basidiomycota presented a trend of decreasing first and then increasing with increasing planting years.

Further analysis of the classification information of species at the genus level indicated that there were great differences between different planting years at the genus level. As shown in Figure 3C, the dominant bacterial genus *Geobacter* tended to first decrease and then increase with increasing planting years. The relative abundance of *Clostridium* genus was highly enriched in Y20, whereas *Gp1* genus was enriched in Y10. In the fungal community (Figure 3D), the relative abundance of *Mortierella* genus increased with increasing planting years. The relative abundances of *Pseudeurotium* and *Neurospora* genus were highly abundant in Y20. *Ustilago* genus was significantly enriched in Y5 but abruptly decreased afterwards. The relative abundances of *Trichoderma* and *Westerdykella* genus were highly enriched in Y10 and Y15, respectively.

### 3.4. Relationships between the Microbial Community and Soil Properties

**Figure 4:**
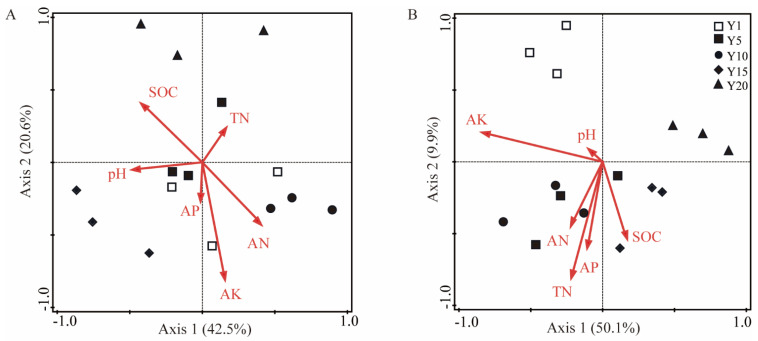
Redundancy analysis of soil properties and community composition of bacteria (**A**) and fungi (**B**). SOC: soil organic carbon; TN: total nitrogen; AN: alkali−hydrolyzable nitrogen; AP: available phosphorus; AK: available potassium. Y1: soil from water oat planting for 1 year. Y5, Y10, Y15 and Y20: soil from continuous water oat replanting for 5, 10, 15 and 20 years, respectively.

RDA was performed on the bacterial and fungal communities and physicochemical properties of the water oat rhizosphere soil of different planting years. As shown in Figure 4, the first and second main axes in the RDA of soil bacteria (Figure 4A) and fungi (Figure 4B) explained 63.1% and 60.0% of the total variation at the phylum level, respectively, which indicated that the first two axes could satisfactorily reflect the relationship between the soil bacterial and fungal community structures and soil chemical properties. The sample points of different years were clearly separated, indicating that planting years had certain effects on the community structures of bacteria and fungi. The AK, AN, and SOC were the main factors that changed the bacterial community structures in soil (Figure 4A), while the AK and AP significantly affected the fungal community structures (Figure 4B).

**Figure 5:**
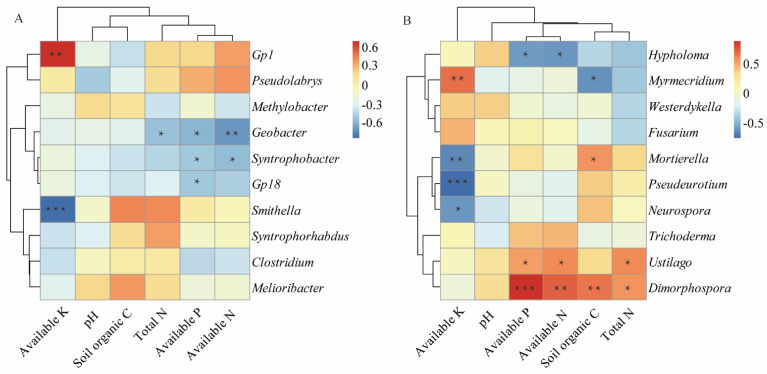
Heatmap of the Spearman correlation analysis between soil variables and the 10 most dominant bacterial (**A**) and fungal (**B**) genera. * *p* < 0.05. ** *p* < 0.01. *** *p* < 0.001.

The Spearman correlation heat map showed that at the bacterial genus level (Figure 5A), *Geobacter* and *Syntrophobacter* significantly or extremely significantly negatively correlated with the AN and AP; *Smithella* extremely significantly negatively correlated with the AK, and *Gp1* extremely significantly positively correlated with the AK. At the fungal genus level (Figure 5B), *Dimorphospora* and *Ustilago* significantly or extremely significantly positively correlated with the AP, AN, and TN, respectively. *Hypholoma* significantly negatively correlated with the AP and AN. *Dimorphospora* and *Mortierella* significantly or extremely significantly positively correlated with the SOC, respectively. *Myrmecridium* significantly negatively correlated with the SOC. *Neurospora*, *Pseudeurotium*, and *Mortierella* significantly or extremely significantly negatively correlated with the AK, but *Myrmecridium* extremely significantly positively correlated with the AK.

## 4. Discussion

Long-term continuous planting leads to an imbalance of soil nutrients, the degeneration of soil functioning and decreased plant yields [26,32]. In this study, the results showed that contents of soil nutrients and the activities of soil enzymes presented the same changing trend with planting years, while the nutrient contents and enzyme activities were much high in Y5 and Y10 but significantly low in Y20 (Table 1 and Figure 1). SOC, AN and AP are essential for soil ecology function and can provide nutrients for growth of plants and microbes [33]. Our results showed that the contents of SOC, TN, AN and AP increased to varying degrees in short-term continuous planting years (Y5 and Y10), which were similar to the findings under three-year continuous monocropping [17]. However, the long-term planting of water oat caused a decrease in soil nutrients. These findings were consistent with the results of Niu et al. (2015) and Chen et al. (2018), who found that soil organic matter, available N and P were significantly reduced after 25 years of continuous cropping [34,35]. In general, soil nutrient contents decrease significantly after long-term continuous replanting, indicating that long-term continuous planting can lead to the partial loss and proportional imbalance of soil nutrients [26]. The activities of sucrase and catalase are closely related to the intensity of SOC conversion and respiration and serves as an important indicator of soil fertility [24]. This study showed that activities of urease, catalase, and phosphatase in soil under continuous cropping of water oat first increased and then decreased. In particular, the enzyme activities increased after 5 and 10 years of continuous planting but decreased after 15 and 20 years. This result is similar to the conclusions of Lou et al. (2007) after studying the activities of catalase, urease, and neutral phosphatase in the soil under different tobacco planting years and that drawn by Nan et al. (2015) when studying the activities of urease and sucrase in alfalfa (*Medicago sativa*) rhizosphere soil [23,25]. Wang et al. (2022) suggested that high increment in the activities of urease, catalase, sucrase and phosphatase under 0–3-year continuous cropping is explained by increases in soil properties and microbial diversity index [17]. In this study, there were significant positive correlations between the activities of four soil enzymes and soil variables including SOC, TN, AN and AP (Table 2). Moreover, the gene abundance and diversity index of fungal community increased in Y5 and then reduced in Y20, which may indicate that water oat under different continuous planting years recruit fungal taxa to affect enzyme production [17].

Soil microbes play a crucial role in maintaining the sustainability of soil functions and ecosystems and are often considered as a sensitive biological indicator of soil health [36,37]. The results of this study showed that there was no significant difference in bacterial abundance between the different planting years, while fungal abundance first increased in Y5 and Y10 and then decreased in Y20. This result is consistent with the findings of studies on other crops, suggesting that short-term fertilization can increase the contents of soil nutrients and promote microbial growth [1,14]. Our results showed that the long-term continuous planting of water oat (Y15 and Y20) significantly reduced the OTU numbers and Shannon, Chao1, and ACE indices of bacterial and fungal communities (Figure 2). These finding are consistent with recent studies on different crop species [38,39], who found that the long-term continuous planting reduced the diversity and abundance of bacteria and fungi in the rhizosphere soil. They suggesting that long-term continuous planting lowered the bacterial and fungal diversity [40]. In long-term continuous planting system, the same type of root exudates is secreted continuously in the rhizosphere, that can stimulate the colonization of certain microbial groups; this may be part of possible reasons for the reduction in rhizosphere microbial diversity under long-term continuous planting [15,41].

The analysis on microbial community structures indicated that Proteobacteria, Chloroflexi, Bacteroidetes, Acidobacteria, and Firmicutes were the dominant bacterial phyla, while Mortierellomycota, Ascomycota, Basidiomycota, and Rozellomycota dominated the fungal community (Figure 3). Proteobacteria is a dominant phylum in different geographical areas and soil types, which has been validated in this study. Pankratov et al. (2011) claimed that the abundance of Acidobacteria was related to soil pH and that a lower soil pH results in higher abundance of Acidobacteria [42]. This study clarified that Acidobacteria first increased and then decreased with continuous planting years and peaked after 10 years of continuous planting (Figure 3A). This study found that the abundance of Acidobacteria was the lowest after 20 years of continuous cropping, suggesting that long-term continuous cropping can lead to an increase in Acidobacteria and pose a potential continuous cropping obstacle [43]. Chloroflexi and Bacteroidetes play vital roles in the degradation of organic matter, carbon and nitrogen cycling [36]. The higher abundance of Bacteroidetes in Y10 might be explained by the production of more litter in the water oat roots. Mortierellomycota and Ascomycota were the dominant phyla in the rhizosphere of water oat (Figure 3B). Our results showed that the relative abundances of Ascomycota and Mortierellomycota in the water oat rhizosphere soil increased significantly after 5–20 years of continuous cropping, which was consistent with the conclusion drawn by Song et al. (2022) under continuous cropping of soybean (*Glycine max*) [44]. Ascomycota is a saprophytic fungus that can rot and decompose litter and is the main source of toxins that cause plant diseases [45]. The accumulation of fungal pathogens is at the expense of beneficial fungi in plants, suggesting that the increase in planting years decreases the numbers of beneficial fungi and increases those of pathogenic bacteria. Therefore, the increase in Ascomycota may raise the risk of disease in water oat. Basidiomycota plays a dominant role in the subsequent decomposition of litter. As a result of its ability to degrade lignin-containing substances, it has a higher relative abundance in environments with better soil quality [46]. This study showed that the relative abundance of Basidiomycota after 10–20 years continuous cropping was lower than that after one- and five-years continuous cropping.

The soil properties are closely related to soil microbial community composition, and long-term continuous cropping leads to the deterioration of soil properties and the imbalance of soil nutrient contents [32]. In this study, the continuous planting of water oat significantly affected soil properties (Table 1). The RDA results showed an obvious separation between the sample plots of different planting years, which indicated that planting years affected the bacterial and fungal community structures to some extent. Furthermore, the AK, AN, and SOC were the main factors that changed the bacterial community structure in rhizosphere soil, while the AK and AP significantly affected the fungal community structure. These results were consistent with the results obtained by Geng et al. (2020) and Liu et al. (2021) [15,47]. In this study, *Geobacter* negatively correlated with the TN, AN, and AP (Figure 5A). The relative abundance of *Geobacter* tended to decrease first and then increase with the continuous planting years (Figure 3C). *Neurospora* and *Pseudeurotium* negatively correlated with the AK (Figure 5B), that were significantly enriched after 20 years of continuous cropping, suggesting that the soil available nutrients play an important role in maintaining the balance of soil bacterial and fungal communities. Our results indicated that there were significant differences in the dominant bacterial and fungal taxa between the long-term and short-term continuous planting years. These findings were consistent with the results obtained by Liu et al. (2021) and Niu et al. (2015) [15,34]. Moreover, it is apparent that soil properties more strongly correlated with the dominant fungal genera than the dominant bacterial genera (Figure 5), suggesting that the change in soil nutrient has a greater effect on soil fungi than bacteria. Clarifying the effects of different continuous planting years on the soil bacterial and fungal communities and exploring their changing trends facilitate the rational arrangement of water oat planting, which is highly significant for the sustainable development of water oat.

## 5. Conclusions

In summary, we found that continuous replating year affected the soil properties and enzyme activities of water oat, with significant differences between short- and long-term planting. The community structure of bacteria and fungi in rhizosphere soil were shifted under continuous replanting years. The diversity of bacterial and fungal communities was decreased after long-term water oat planting (Y15 and Y20). The fungal abundance was high under continuous replanting of water oat, but no change in bacterial abundance was observed between the different replanting years. In addition, the AK, AN, and SOC were the main factors that changed the bacterial community, while AK and AP significantly shifted the fungal community structure. Based on the above results, we speculate that the microbial community and function in rhizosphere soil of water oat depend on the dynamic changes in soil nutrients. The results provided a reference for the remediation of soil under continuous water oat planting and the sustainable development of the water oat industry.

## Figures and Tables

**Table 1:** Soil properties and microbial gene abundances in soil under different planting years of water oat.

Treatment	pH	SOC (g·kg^−1^)	TN (g·kg^−1^)	AN (mg·kg^−1^)	AP (mg·kg^−1^)	AK (mg·kg^−1^)	Bacterial Abundance (×10^11^ Copies·g^−1^ Soil)	Fungal Abundance (×10^9^ Copies·g^−1^ Soil)
Y1	5.65 ± 0.29 ^a^	23.93 ± 0.72 ^b^	2.17 ± 0.28 ^b^	138.10 ± 9.54 ^c^	32.02 ± 3.62 ^c^	170.24 ± 17.40 ^a^	2.92 ± 0.31 ^a^	1.25 ± 0.54 ^b^
Y5	5.69 ± 0.02 ^a^	32.17 ± 2.40 ^a^	2.97 ± 0.38 ^a^	177.28 ± 10.09 ^b^	67.74 ± 7.71 ^a^	123.67 ± 16.13 ^b^	2.03 ± 0.35 ^a^	2.40 ± 0.45 ^ab^
Y10	5.46 ± 0.29 ^a^	28.73 ± 1.80 ^ab^	2.85 ± 0.24 ^a^	195.33 ± 11.61 ^a^	72.04 ± 4.81 ^a^	131.62 ± 16.56 ^b^	2.91 ± 0.54 ^a^	3.01 ± 0.56 ^a^
Y15	5.71 ± 0.07 ^a^	30.84 ± 5.27 ^ab^	2.56 ± 0.33 ^ab^	170.61 ± 9.57 ^b^	69.41 ± 6.59 ^a^	115.68 ± 4.61 ^b^	2.42 ± 0.40 ^a^	2.83 ± 0.29 ^a^
Y20	5.56 ± 0.23 ^a^	30.11 ± 1.44 ^ab^	2.56 ± 0.04 ^ab^	144.44 ± 7.52 ^c^	44.05 ± 1.80 ^b^	31.85 ± 2.30 ^c^	2.28 ± 0.33 ^a^	1.83 ± 0.61 ^ab^

SOC: soil organic carbon; TN: total nitrogen; AN: alkali−hydrolyzable nitrogen; AP: available phosphorus; AK: available potassium. Y1: soil from water oat planting for 1 year. Y5, Y10, Y15 and Y20: soil from continuous water oat replanting for 5, 10, 15 and 20 years, respectively. Data are presented as the mean ± standard error (*n* = 3). Different letters indicate significant differences among the treatments at the *p* < 0.05.

**Table 2:** Correlations between soil properties and enzyme activities in soil under different planting years of water oat.

	pH	SOC	TN	AP	AK	AN
Urease	−0.052	0.384	0.778 **	0.722 **	−0.216	0.726 **
Sucrase	0.065	0.525 *	0.586 *	0.586 *	−0.230	0.689 **
Catalase	0.011	0.582 *	0.842 ***	0.836 ***	−0.153	0.836 ***
Acid phosphatase	−0.090	0.314	0.692 **	0.836 ***	0.039	0.914 ***

SOC: soil organic carbon; TN: total nitrogen; AN: alkali-hydrolyzable nitrogen; AP: available phosphorus; AK: available potassium. * *p* < 0.05. ** *p* < 0.01. *** *p* < 0.001.

## Data Availability

We have deposited all raw sequences in National Center for Biotechnology Information (NCBI) Sequence Reads Archive database under accession number PRJNA883442.

## Abbreviations

qPCR, quantitative real-time PCR; OTU, operational taxonomic unit; RDA, redundancy analysis; ITS, internal transcribed spacer.
